# The efficacy and safety of risperidone combined with modified electroconvulsive therapy in the treatment of schizophrenia

**DOI:** 10.12669/pjms.41.10.12716

**Published:** 2025-10

**Authors:** Rui Zhang, Wenzhu Yin, Zhichao Wang

**Affiliations:** 1 Rui Zhang, Department of Sleep Ward, Heilongjiang Provincial Third Hospital, Beian, Heilongjiang Province 164092, P.R. China; 2 Wenzhu Yin, Department of Sleep Ward, Heilongjiang Provincial Third Hospital, Beian, Heilongjiang Province 164092, P.R. China; 3 Zhichao Wang, Department of Psychiatry, Heilongjiang Provincial Third Hospital, Beian, Heilongjiang Province 164092, P.R. China

**Keywords:** Adverse events, Efficacy, Modified electroconvulsive therapy, Risperidone, Schizophrenia

## Abstract

**Objective:**

To explore the efficacy and safety of risperidone combined with modified electroconvulsive therapy (MECT) in the treatment of schizophrenia.

**Methodology:**

Clinical records of 180 patients with schizophrenia, treated at the Third Hospital of Heilongjiang Province from June 2022 to October 2024, were retrospectively analyzed. The cohort included 90 patients treated with risperidone combined with MECT matched to a cohort treated with risperidone alone in a 1:1 ratio. The main outcome was the change in Positive and Negative Syndrome Scale (PANSS) score. Cognitive function, cytokine levels, and adverse events (AEs) before and after treatment were evaluated.

**Results:**

After treatment, the scores of PANSS general psychopathology, PANSS-positive, and PANSS-negative in both groups decreased compared to before treatment, and were significantly lower in patients receiving risperidone combined with MECT than in patients receiving risperidone alone (P<0.05). The combined treatment was associated with lower non-perseverative errors and higher correct responses than risperidone monotherapy (P<0.05). The levels of serum glial fibrillary acidic protein (GFAP) in both groups decreased compared to before treatment. In contrast, the levels of brain-derived neurotrophic factor (BDNF) increased compared to before treatment, and were higher in patients receiving the combined treatment compared to the risperidone monotherapy group (P<0.05). There was no statistically significant difference in the incidence or total number of AEs between the two groups (P>0.05)

**Conclusions:**

Compared with the use of risperidone alone, the combination of risperidone and MECT is more effective in treating schizophrenia, with no increase in AEs.

## INTRODUCTION

Schizophrenia is characterized by cognitive impairment, positive and negative symptoms.^1^,^2^ Patients often have varying degrees of social dysfunction, which imposes a significant burden on both their families and society.^1^-^3^ Second-generation antipsychotic drugs, such as risperidone, have become commonly used in clinical practice to treat schizophrenia patients.^4^,^5^ Risperidone can regulate the expression of neurotransmitters such as 5-hydroxytryptamine and dopamine to alleviate clinical symptoms.^5^ However, the drug treatment cycle is relatively long, is associated with adverse effects such as mental fatigue and obesity, and is prone to relapse after discontinuation, leading to limitations in its clinical application.^4^-^6^

Electroconvulsive therapy, including modified electroconvulsive therapy (MECT), is widely recognized as an effective method for treating mental disorders.^7^ MECT relies on injecting an appropriate amount of muscle relaxant during the treatment process with general anesthesia and a short-acting muscle relaxant to attenuate motor manifestations of the seizure and improve tolerability.^7^,^8^ This technique has become a highly accepted treatment method for patients with schizophrenia.^7^-^9^ In recent years, the effectiveness of combining pharmaceuticals with MECT in treating mental disorders has become a focus of research.^10^,^11^ Bai et al.^10^ confirmed that the combination of conventional drugs and MECT has a significant effect on the treatment of schizophrenia. Tao et al.^11^ found that combining risperidone and aripiprazole with the MECT treatment for schizophrenia is more beneficial for improving and promoting patients’ memory and cognitive function.

Although numerous studies^10^-^12^ have confirmed the effectiveness and safety of the combined approach, there is limited research on the efficacy and safety of single risperidone combined with MECT in the treatment of schizophrenia. This study aimed to evaluate the effectiveness and safety of risperidone combined with MECT in the treatment of schizophrenia, to provide a practical reference for disease treatment.

## METHODOLOGY

This retrospective cohort study included schizophrenia patients who received single risperidone or risperidone combined with MECT in Third Hospital of Heilongjiang Province from June 2022 to October 2024. According to age, gender, body mass index (BMI), disease duration, educational level, family history of schizophrenia, and marital status as matching conditions, patients were allocated in a 1:1 ratio to the Risperidone group and Risperidone & MECT group.

### Ethical approval:

The study protocol was approved by the Ethics Committee of the Third Hospital of Heilongjiang Province (approval No. 2025012; June 16, 2025). Given the retrospective design and use of anonymized clinical data, the requirement for informed consent was waived in accordance with national regulations and the Declaration of Helsinki.

### Inclusion criteria:

- Meets the diagnostic criteria for schizophrenia.^1^
- Age range: 18-65 years old.
- Positive and Negative Symptom Scale (PANSS) score ≥ 60 points.^13^
- Received continuous treatment for more than three months.
- Complete clinical data.

### Exclusion criteria:

- History of other mental system disorders or neurodegenerative diseases (mood disorders, anxiety disorders, obsessive-compulsive disorder, post-traumatic stress disorder, somatoform disorders, mental retardation, and organic mental disorders, etc.).
- Received transcranial magnetic stimulation (TMS) treatment.
- History of drug abuse or dependence (including alcohol and illicit substances; heavy nicotine use was recorded separately).
- Patients with comorbid malignant tumors.
- Serious physical illness.
- Individuals with severe organic brain diseases.
- Severe cardiovascular disease.
- Breastfeeding and pregnant women.

### Risperidone group:

Risperidone (specification: 1mg/tablet, Yangsen Pharmaceutical Co., Ltd., Xi’an, China) was administered at the initial dose of 1 mg/day, increased by 1 mg every 1–2 days. The treatment dose was adjusted to 4-6 mg within two weeks, with a maximum daily drug dose of ≤ 6 mg.

### Risperidone & MECT group:

MECT was done based on the risperidone treatment. Fasting and water restriction were required six hours before treatment, and the patient was required to empty the bowels and bladder. Under the routine monitoring of vital signs, intravenous injection of 2-4 mg/kg propofol (Jiangsu Nhwa Pharmaceutical Co., Ltd, China) and 0.5 mg atropine (Henan Runhong Pharmaceutical Co., Ltd, China) was initiated before treatment. After the eyelash reflex disappeared and the eyeball was fixed, intravenous injection of 50-80 mg of succinylcholine chloride (0.2%) (Xi’an Hanfeng Pharmaceutical Co., Ltd. China) was administered. When the patient’s breathing got shallower or slower, an oxygen mask was used for artificial respiration. After the tremor of the limb muscle bundle ended, the oxygen mask was removed, and an oral protector was inserted before starting MECT. The Thymatron System IV electrodes were placed on both sides of the forehead. Device settings were standardized as follows: impedance <1,500 Ω, pulse frequency 30 Hz, and pulse-train duration three seconds. The initial stimulus charge was estimated using the half-age method and was subsequently titrated by 5–10% between sessions according to seizure adequacy and clinical response. MECT administration schedule: Treatments were delivered three times per week (Monday/Wednesday/Friday) at 48–72 hours intervals during the acute phase. Each patient received a total of 8–12 sessions (median 10; IQR 9–12) over approximately 3–4 weeks (median 3.5 weeks). No maintenance ECT was administered during the study period.

The main outcome was symptom relief, evaluated based on the Positive and Negative Symptom Scale (PANSS). Assessment included a total score of 210 points, 7-item PANSS positive (max 49), 7-item PANSS negative (max 49), and 16-item PANSS general psychopathology (max 112), with a higher score indicating more severe schizophrenia.^13^ Secondary outcomes were as follows:

***Cognitive function measurement***. Assessment was done according to the Wisconsin Card Sorting Test (WCST) evaluation, with 128 cards. The score ranged 0-128 points and took into account the number of categories completed, non-perseverative errors, perseverative errors, and correct responses. Better cognitive function was indicated by more categories completed and correct responses, and fewer perseverative and non-perseverative errors.

***Biomarker measurements (GFAP and BDNF)***. Fasting venous blood (4 mL) was collected, allowed to clot at room temperature for 30 min, and centrifuged at 1,500 × g for 10–15 min. Serum was aliquoted and stored at −80 °C until analysis, avoiding more than one freeze–thaw cycle. GFAP and BDNF concentrations were determined using double-antibody sandwich ELISA kits (Wuhan BOSTER Biotechnology Co., Ltd., Wuhan, China) according to the manufacturer’s instructions. Samples, standards, and quality controls were assayed in duplicate, preferably on the same plate, and read at 450 nm with a 570 nm reference. Calibration curves were generated using a four-parameter logistic model after blank subtraction, with acceptable back-calculated concentrations within ±15% of nominal values (±20% at the LLOQ). Samples exceeding a duplicate CV of 15% or falling outside the reportable range were re-assayed; dilutions (1:2–1:5) were performed when required, and results multiplied by the dilution factor. Data analysis was blinded to treatment allocation. For GFAP, the LOD was 0.05 ng/mL, LLOQ 0.156 ng/mL, ULOQ 10.0 ng/mL, and the standard curve range 0.156–10.0 ng/mL (eight standards). Intra- and inter-assay CVs were ≤ 8% (median 4.8%) and ≤ 12% (median 7.1%), respectively; spike-recovery was 92–105% (0.8 and 4.0 ng/mL), and dilution linearity r² ≥ 0.995. Median serum GFAP values (1.5–2.7 ng/mL) fell within the quantifiable range, so routine dilution was unnecessary. For BDNF, the LOD was 0.20 ng/mL, LLOQ 1.0 ng/mL, ULOQ 80.0 ng/mL, and the standard curve range 1.0–80.0 ng/mL (1, 2, 5, 10, 20, 40, 80 ng/mL). Intra- and inter-assay CVs were ≤ 8% (median 5.2%) and ≤ 12% (median 8.6%), respectively; spike-recovery was 90–108% (5 and 20 ng/mL), and dilution linearity r²≥0.995. Median serum BDNF concentrations (19–28 ng/mL) were within the quantifiable range; a 1:2 dilution was applied for high-range samples to minimize matrix effects. Plates were accepted only if QC results were within predefined ranges and the calibration curve fit was r²≥0.99.

The occurrence of AEs, including transient headache, constipation, dizziness, memory impairment, nausea and vomiting, etc.

### Adherence monitoring and discontinuation:

Treatment adherence was monitored throughout the index treatment period by reviewing medical records, daily nursing logs, and medication administration charts. For MECT, adherence was verified using attendance logs for each scheduled session. Premature discontinuation was defined a priori as stopping risperidone for ≥7 consecutive days or missing ≥2 consecutive MECT sessions; poor adherence was defined as taking <80% of prescribed risperidone doses or attending <80% of scheduled MECT sessions. No protocol-mandated rescue changes were allowed during the index period.

### Statistical analysis:

Statistical analyses were performed in SPSS 25.0 (IBM, Armonk, NY). Data distributions were evaluated using the Shapiro–Wilk test, and homogeneity of variances using Levene’s test. For between-group comparisons of continuous variables, we used the Student’s t-test when normality and homoscedasticity were satisfied, and the Wilcoxon rank-sum test otherwise. For within-group (pre/post) comparisons, we used the paired t-test when assumptions held and the Wilcoxon signed-rank test otherwise. Categorical variables were compared using the chi-square test or Fisher’s exact test, as appropriate. All tests were two-tailed with α=0.05. Adherence and discontinuation outcomes were summarized descriptively.

## RESULTS

This study included 180 patients, with 91 males, accounting for 50.6% (91/180). The age range was 18-65 years, with a median of 42 (33.5-52) years. Patients in the Risperidone & MECT and the Risperidone group were matched in a 1:1 ratio, 90 patients in each group. There was no statistically significant difference in baseline characteristics between the two groups (P>0.05) (Table-I).

**Table-I:** Comparison of baseline characteristics between two groups.

Characteristics	Risperidone & MECT (n=90)	Risperidone (n=90)	Z	P
Age (years),M(IQR)	42 (35-51)	45 (32-54)	-0.259	0.796
Male (yes), n(%)	47 (52.2)	44 (48.9)	0.200	0.655
BMI(kg/m²),M(IQR)	25.2 (23.6-26.4)	25.75 (23.6-28.2)	-1.538	0.124
Disease duration (years), M(IQR)	5 (4-8)	6 (5-8)	-1.732	0.083
Educational level, n(%)			0.415	0.520
Junior high school and below	60 (66.7)	64 (71.1)		
High school and above	30 (33.3)	26 (28.9)		
Family history (yes), n(%)	25 (27.8)	30 (33.3)	0.655	0.418
Marital status, n(%)			3.047	0.384
Unmarried	38 (42.2)	33 (36.7)		
Married	38 (42.2)	35 (38.9)		
Divorced	12 (13.3)	16 (17.8)		
Widowed	2 (2.3)	6 (6.7)		

No participants discontinued risperidone or MECT prematurely, and no cases met the predefined criteria for poor adherence. According to medication administration charts, in-hospital risperidone adherence was 100%. Attendance logs indicated that all patients allocated to the Risperidone & MECT group completed the planned MECT course (8–12 sessions over approximately 3–4 weeks) without missed sessions.

In terms of the main outcome, there was no significant difference in the scores of PANSS general psychopathology, PANSS-positive, and PANSS-negative between the two groups before treatment (P>0.05). After treatment, the scores of PANSS general psychopathology, PANSS-positive, and PANSS-negative in both groups decreased compared to before treatment, and were considerably lower in the Risperidone & MECT group than in the Risperidone group (P<0.05) (Table-II).

**Table-II:** Comparison of the Positive and Negative Symptom Scale (PANSS) score between the two groups before and after treatment, M(IQR).

Variables	Risperidone & MECT (n=90)	Risperidone (n=90)	Z	P
***Before intervention***				
PANSS-general psychopathology score	40 (28-51)	41 (33-55)	-0.674	0.500
PANSS-positive score	24 (22-26)	23 (20-26)	-1.819	0.069
PANSS-negative score	22 (21-26)	24 (21-27)	-1.744	0.081
Total score	86 (75-97)	88 (79-102)	-0.780	0.435
***After intervention***				
PANSS-general psychopathology score	19.5 (18-23)^#^	23 (21-27)^#^	-4.936	<0.001
PANSS-positive score	16 (14-19)^#^	19.5 (16-21)^#^	-4.763	<0.001
PANSS-negative score	16 (13-18)^#^	18.5 (16-23)^#^	-3.421	0.001
Total score	53 (49-58)^#^	62 (58-68)^#^	-6.901	<0.001

Compared with before treatment in the same group,

# P<0.05.

Before treatment, there was no significant difference in the number of categories completed, non-perseverative errors, perseverative errors, and correct responses between the two groups (P>0.05). After treatment, both groups showed an increase in the number of categories completed and correct responses compared to before treatment, while the scores for non-perseverative errors and perseverative errors decreased (P<0.05). The number of non-perseverative errors in the Risperidone & MECT group was lower, and the number of correct responses was higher than that in the Risperidone group (P<0.05) (Table-III). These WCST changes—fewer non-perseverative errors and more correct responses—are consistent with improved executive control (cognitive flexibility, working memory updating, and sustained attention).

**Table-III:** Comparison of the Wisconsin Card Sorting Test (WCST) between the two groups before and after treatment, M(IQR).

Variables	Risperidone & MECT (n=90)	Risperidone (n=90)	Z	P
Before intervention				
Categories completed	3 (2-3)	3 (2-4)	-1.072	0.284
Non perseverative errors	28.5 (25-34)	26 (24-34)	-0.162	0.105
Perseverative errors	27 (24-33)	25 (23-33)	-1.840	0.066
Correct responses	28 (22-32)	31 (23-33)	-1.634	0.102
After intervention				
Categories completed	5 (4-5)	4 (3-5)	-1.532	0.126
Non perseverative errors	22.5 (19-29)^#^	25 (21-29)^#^	-2.135	0.033
Perseverative errors	21 (18-27)	23 (19-25)	-1.487	0.137
Correct responses	35 (29-39)^#^	34 (26-36)^#^	-2.484	0.013

Compared with before treatment in the same group,

# P<0.05.

Before treatment, the two groups had no significant difference in serum GFAP and BDNF levels (P>0.05). After treatment, the serum GFAP levels in both groups decreased compared to before treatment, and were considerably lower in the Risperidone & MECT group. In contrast, the level of BDNF increased compared to before treatment, and was markedly higher in the Risperidone & MECT group compared to the Risperidone group (P<0.05) (Table-IV). The incidence and the total number of AEs were comparable in the two groups (P>0.05) (Table-V).

**Table-IV:** Comparison of neurotrophic factors between the two groups, M(IQR).

Variables	Risperidone & MECT (n=90)	Risperidone (n=90)	Z	P
***Before intervention***				
GFAP (ng/ml)	2.59 (2.35-3.2)	2.71 (2.42-3.46)	-1.298	0.194
BDNF (ng/ml)	17.8 (15.2-22.7)	19.3 (15.3-24.2)	-1.116	0.264
***After intervention***				
GFAP (ng/ml)	1.54 (1.32-2.11)	2.12 (1.83-2.79)	-5.187	<0.001
BDNF (ng/ml)	27.7 (24.9-32.7)	26.15 (22.5-31.7)	-2.235	0.025

**Table-V:** The occurrence of adverse events (AEs) in two groups, n(%).

AEs	Risperidone & MECT (n=90)	Risperidone (n=90)	χ^2^	P
Transient headache	6 (6.7)	2 (2.2)		0.148^b^
Constipation	6 (6.7)	1 (1.1)		0.123^b^
Dizziness	5 (5.6)	3 (3.3)		0.718^b^
Memory impairment	5 (5.6)	1 (1.1)		0.441^b^
Nausea and vomiting	7 (7.8)	10 (10.0)	0.274	0.600^a^
Total number of occurrences	24 (26.7)	16 (17.8)	2.057	0.151^a^

a Pearson’s Chi-square test;

b Fisher’s Exact Test.

## DISCUSSION

This study demonstrated that risperidone combined with MECT was associated with greater improvement across PANSS domains and WCST performance compared with risperidone alone, while the overall safety profile was comparable between groups (P>0.05). This study is consistent with the previous results of Yang et al.^14^ and Chen et al.^15^ and further confirms that the combination of risperidone and MECT can improve the symptoms and cognitive function of patients with schizophrenia. Such an effect can be explained by the synergistic action of the two components: risperidone can reduce excessive dopamine activity in the midbrain limbic system, thereby alleviating hallucinations and delusions, while MECT can induce electrophysiological changes in the brain, affecting the release of neurotransmitters and neuronal plasticity.^14^,^15^

Importantly, the WCST pattern observed in our cohort—greater correct responses alongside fewer non-perseverative errors—maps onto core executive functions, including cognitive flexibility (set-shifting), problem-solving/goal maintenance, working memory updating, and sustained attention. In real-world terms, gains in these domains are expected to facilitate day-to-day problem solving (e.g., planning and executing multi-step tasks), adaptive strategy shifting when routines or rules change (e.g., ward schedules or workplace demands), and more consistent engagement in rehabilitation, vocational, and social activities.^16^ Prior literature has linked WCST performance with functional capacity and community participation in schizophrenia;^17^,^18^ thus, beyond symptom reduction, our data suggest that risperidone plus MECT may translate into meaningful improvements in executive-function–dependent daily functioning that merit prospective confirmation.

The results of this study showed that the combination of risperidone and MECT can better improve the levels of GFAP and BDNF in patients with schizophrenia. This is consistent with the research results of Xu et al.^17^ and Szota et al.^18^ In schizophrenia patients, GFAP serves as a biomarker of abnormal activation of glial cells.^17^,^19^ Studies also show that schizophrenia is associated with lower levels of BDNF, which plays a crucial role in the survival, differentiation, and synaptic plasticity of neurons.^18^,^20^ Risperidone and MECT can inhibit the overactivation of glial cells through different mechanisms. Risperidone can indirectly affect the function of glial cells by regulating the neurotransmitter system, while MECT can directly act on brain cells, reducing the stress response of glial cells and thus lowering GFAP levels.^17^,^18^ At the same time, combination therapy can more effectively stimulate endogenous BDNF production in the brain. The neurophysiological changes caused by MECT can promote the release of BDNF from neurons, while risperidone maintains this elevated state, thereby playing a positive role in neuronal repair and functional improvement.^17^,^18^,^20^

While MECT is a commonly used psychiatric treatment method with significant advantages in improving effectiveness and expanding indications, this method still has limitations. Although electrical stimulation methods are gradually being optimized and improved, some patients may still experience discomfort, such as headaches, constipation, dizziness, memory impairment, nausea and vomiting.^21^ This study’s results showed no statistically significant difference in the total number of AEs between the two groups. However, 26.7% of the patients in the Risperidone & MECT group still experienced AEs, somewhat higher than the 17.8% in the Risperidone group. These results highlight a need to reduce the incidence of AEs further.^21^ From a mechanistic perspective, a slightly higher frequency of mild AEs in the MECT group can be plausibly attributed to both neurophysiological and anesthetic factors.^22^ Brief electrical stimulation under general anesthesia transiently increases cortical excitability and induces widespread neuronal depolarization, accompanied by rapid shifts in glutamatergic and GABAergic transmission and short-lived changes in cerebral blood flow and metabolism.^23^ These acute perturbations may manifest as post-ictal headache, dizziness, and transient cognitive inefficiency in susceptible patients. In addition, periprocedural medications can contribute to self-limited AEs: propofol may cause short episodes of hypotension or apnea despite its antiemetic and anticonvulsant properties; succinylcholine can produce myalgia and rare autonomic fluctuations; and anticholinergic premedication (e.g., atropine) may increase the likelihood of constipation or transient tachycardia.^24^ Importantly, in our cohort these events were mild to moderate, time-limited, and resolved with routine monitoring and supportive care, which aligns with the overall comparable AE burden between groups.

Therefore, future research should focus on exploring the use of anesthetic drugs while maintaining sedative effects in MECT treatment. In addition, Martin et al.^24^ suggested that adjusting pulse width may help reduce cognitive impairment in patients. Memory impairment is reversible, and with neural repair, as the condition improves, memory function will improve. Yang et al.^25^ found that MECT is a risk factor for hospital-acquired pneumonia in patients with schizophrenia. However, no pneumonia was observed in this study, which may be related to the sample size. It is currently unclear whether these adverse reactions are related to the patient’s physical illness. Therefore, there is a need to screen patients before administering the combined risperidone/MECT treatment to clarify whether the patient meets the treatment principles for MECT.^24^-^26^ Individualized treatment measures should be taken for different patients, providing psychological support and improving treatment compliance.

Our matched cohort provides integrated evidence across symptoms, cognition, and neurobiological markers for the specific combination of risperidone plus MECT. Median PANSS total improved from 86 (IQR 75–97) to 53 (49–58) with combination therapy versus 88 (79–102) to 62 (58–68) with risperidone alone, and WCST showed fewer non-perseverative errors and more correct responses after combination therapy. In parallel, serum GFAP decreased and BDNF increased more prominently in the combination group, suggesting astroglial dampening and neurotrophic support that may underlie clinical gains. Notably, these benefits were achieved without a statistically significant increase in adverse events (26.7% vs. 17.8%, P=0.151), supporting the clinical applicability of adding MECT to risperidone under routine monitoring. Strengths of this study include: (i) a 1:1 matched design on key baseline variables (age, sex, BMI, duration, education, family history, marital status) improving internal comparability; (ii) standardized MECT delivery (device, electrode placement, impedance <1500 Ω, 30 Hz, stimulus duration three seconds, age-based dosing titration) enhancing reproducibility; (iii) multidimensional outcomes encompassing PANSS, WCST, and serum GFAP/BDNF, linking clinical change with plausible biological mechanisms; and (iv) complete on-protocol adherence to risperidone and scheduled MECT sessions during the index course, minimizing bias from treatment discontinuation.

### Limitations:

First, it was a single-center retrospective analysis with a relatively small sample size, which may limit the generalizability of the findings. Second, to minimize confounding, only patients aged 18–65 years were included, potentially restricting applicability to younger or older populations. Third, the frequency of MECT sessions was fixed without individualized adjustment, which might increase the risk of unnecessary side effects in patients who could benefit from lower treatment intensity. Finally, high-quality, multi-center trials with larger and more diverse cohorts and extended follow-up are warranted to validate the long-term effects of risperidone combined with MECT on cognitive outcomes, cardiovascular safety, and other clinical endpoints. Future research should also evaluate relapse prevention, functional recovery, and the durability of cognitive improvements, incorporate predefined subgroup analyses (e.g., negative-symptom-dominant vs. non-dominant profiles, treatment-resistant status, and age strata) to explore heterogeneity of treatment response, and integrate mechanistic investigations linking seizure parameters, anesthesia regimens, and longitudinal GFAP/BDNF trajectories. Pragmatic trials are further needed to assess cost-effectiveness and real-world implementation feasibility.

## CONCLUSION

Compared with risperidone monotherapy, the combination of risperidone and MECT demonstrated superior efficacy in alleviating psychotic symptoms, enhancing cognitive performance, and modulating serum neurotrophic factor expression, without an increase in adverse events. These findings support the potential value of integrating MECT into treatment regimens for schizophrenia under appropriate clinical monitoring. Further research is warranted to elucidate the underlying mechanisms, determine the durability of therapeutic benefits, and develop individualized treatment strategies. Notably, the observed improvements in WCST performance suggest possible gains in executive-function–dependent daily functioning. Future prospective trials should incorporate standardized functional outcome measures, such as functional capacity and vocational engagement, to validate the real-world applicability of these cognitive enhancements.

### Authors’ contributions:

**RZ:** Literature search, study design and manuscript writing. **WY and ZW:** Data collection, data analysis and interpretation. Critical Review. **RZ:** Critical analysis, manuscript revision and validation and is responsible for the integrity of the study. All authors have read and approved the final manuscript.
